# Phytochemical and Biological Investigation of *Clivia nobilis* Flowers Cultivated in Egypt

**Published:** 2016

**Authors:** Eman Shawky

**Affiliations:** Faculty of Pharmacy, Pharmacognosy department, University of Alexandria, Egypt, Alexandria 21521, Egypt.

**Keywords:** Amaryllidaceae, alkaloids, *Clivia nobilis* flowers, antimicrobial

## Abstract

Amaryllidaceae is a well-known family for its high alkaloidal content. These alkaloids comprise a unique group of bases that have been found to occur in this family. The Amaryllidaceae alkaloids represent a large and still expanding group of isoquinoline alkaloids, the majority of which are not known to occur in any other family of plants. This article reports on the phytochemical investigation of the alkaloidal content of the flowers of *clivia nobilis* cultivated in Egypt which resulted in the isolation of four alkaloids; lycorine with pyrrolo{de}phenanthridine nucleus (lycorine-type) which is the common alkaloid of the amaryllidaceae family, clivatine and nobilisine, both with [2]benzopyrano (3,4-g) indole nucleus (lycorenine-type) and (+) 8-*O*-demethylmaritidine with 5,10b-ethanophenanthridine nucleus (crinine-type). Furthermore, the antimicrobial activity of the chloroform extract of the flowers of *c. nobilis* along with some of the isolated alkaloids has been studied.

## Introduction

Amaryllidaceae is a widely reputable family for its high alkaloidal content. The alkaloids from the extracts of Amaryllidaceae plants have been the object of active chemical investigation for nearly 200 years (1). A particular characteristic of Amaryllidaceae is a consistent presence of an exclusive group of alkaloids, which have been isolated from the plants of all the genera of this family. Plants of the Amaryllidaceae family have been used for thousands of years as herbal remedies. Over the past three decades many have been isolated, screened for different biological activities, and synthesized by a number of research groups. At present, over 300 alkaloids have been isolated from plants of this family (2) and, although their structures vary considerably, these alkaloids are considered to be biogenetically related. Their highly particular skeleton arrangements and broad spectrum of biological activities have prompted numerous chemical and pharmacological studies of this group of alkaloids. Until now only galathamine is being marketed, but the significant activities of other alkaloids in the family demonstrated in recent years could favour their therapeutic use in the near future (3). 

Genus *Clivia* comprises cultivated ornamental perennial herbs. The two species *C. miniata a*nd *C. nobilis* are used in folk medicine in their native countries. The Clivia genus is endemic to South Africa and the most common species, *C. miniata*, is used by traditional healers to facilitate childbirth and as a snake bite remedy (4). Leven *et al. *stated that the folkloric use of *Clivia* is due to its antiviral activity (5, 6). *Clivia* species have been the major source of alkaloids represented by the 3a, 4-dihydro-Lactone(2) benzopyrano[3,4-g]indole ring system and containing four chiral centers at the ring junction positions (3a, 5a11b and 11c) (7). 

The work presented in this article includes phytochemical investigation of the alkaloidal content of the flowers of *C. nobilis* cultivated in Egypt. The reported attributes of this plant and the fact that there is no documented antimicrobial effect necessitated the need to separate, purify, isolate and identify the alkaloids present and also investigate the antimicrobial activity of the extract of the flower as no previous work has been done on *C. nobilis* flowers.

## Experinental

General: Mps. were determined on a Stuart SMP heating stage microscope and are uncorrected. UV spectra were determined on Pye Unicam SP8-100 UV/VIS Spectrophotometer. 1D-NMR and 2D-NMR (COSY, HSQC and HMBC) spectra were recorded at JEOL JNM A-500 (^1^H: 500 MHz, ^13^C: 125 MHz). EI MS were taken at JEOL JMS GC mate. Analytical and preparative TLC were performed on silica gel (Merck, Kieselgel, 60 F_254_, 0.25 and 0.50 mm, respectively). Spots were visualized by exposure to NH_3_ vapour, UV radiation, anisaldehyde/H_2_SO_4_ and Dragendorff’s spray reagents. Authentic reference materials; (+) 8-*O*-demethylmaritidine, lycorine, nobilisine, and clivatine, were stock samples at the Department of Pharmacognosy, Faculty of Pharmacy, University of Alexandria. 

Plant material: *Clivia nobilis*. flowers were collected in August, 2012, cultivated in Alexandria, Egypt. The plant was kindly identified by Professor Alam El-Din Noah (Professor of Ornamental Plantst, Faculty of Agriculture, Alexandria University, Egypt. A voucher sample is deposited in the Department of Pharmacognosy, Faculty of Pharmacy, Alexandria, Egypt.

*Extraction and isolation:*

*Isolation of alkaloids 1-4:*

 Freshly chopped flowers (700 g) were exhaustively extracted with EtOH by maceration. The combined extracts were concentrated under reduced pressure then defatted with pet. ether, acidified with 5% tartaric acid to pH 2, filtered and then washed with Et_2_O. The acidic aqueous phase was rendered alkaline with NH_4_OH solution to pH 10, and then extracted successively with CHCl_3_, EtOAc and *n*-BuOH. The CHCl_3 _­extracts were combined and concentrated to a small volume, at this stage a white residue (0.1 g) was precipitated and identified as lycorine (1) by comparison against a reference sample and filtered out. The filterate was evaporated under reduced pressure to give a residue (0.7 g), which was fractionated over a silica gel column. Elution was started by chloroform, increasing the polarity with methanol. Fractions (100 mL. each) were collected and monitored by TLC (solvent systems 9:1 and 8:2, chloroform: methanol). Chromatographic separation resulted in the isolation of clivatine (2), nobilisine (3) and (+) 8-*O*-demethylmaritidine (4) (11, 8, 5 mg, respectively) identified by using the available spectral data together with comparison with reference alkaloidal samples (Co-chromatography and m.m.p). 

*Antibacterial activity of the petroleum ether extract of C. nobilis flowers:*

Antibacterial and antifungal screenings were carried out using the agar diffusion technique (8) against a Gram-positive bacterium *Staphylococcus aureus* and one Gram-negative bacteria, *Pseudomonas aeroginosea*. Antibacterial activity was carried out by Neamat Hanem Moustafa Dorra, Phd student at the High Institute of Public Health. 

*Agar well diffusion assay:*

About one to two isolated colonies of each of the tested bacterial strains growing on a blood agar plate were inoculated in a tube of sterile DW, and then the broth was matched with 0.5 McFarland turbidity standards. The bacterial suspensions were agitated a vortex mixer immediately prior to use. A sterile cotton swab (on a wooden applicator stick) was dipped into the standardized bacterial suspension. Broth was expressed from the swabs by pressing and rotating the swabs firmly against the inside of the tube above the fluid level. The swab was then evenly streaked in three directions over the entire surface of the agar plate to obtain uniform inoculums; a final sweep of the agar rim was made with the cotton swabs. Wells were made on Muller Hinton agar plates using a sterile borer. The plates were allowed to dry for 3–5 minutes after which 100µL of the test samples were dispensed into each well. The concentration of the test samples were 1 mg/mL. The plates were incubated at 37 °C for 24 h during which activity was evidenced by the presence of a zone of inhibition surrounding the well. Zone sizes were measured in millimeters compared to standard ciprofloxacin for Gram negative and Amoxicillin/clavulinic acid for Gram positive.

*Broth dilution method for determination of MIC and MBC*: 

The bacterial suspension was prepared as follow: One to two isolated colonies of tested organisms were picked by sterile inoculating loop and inoculated in a tube of sterile DW (5 mL) then matched with 0.5 McFarland turbidity standards. Equal volumes of each bacterial isolate culture (100µL) containing approximately 10^8 ^CFU/mL were applied onto nutrient broth (2 mL) supplemented by the herbal extract with concentrations of 1000, 500, 250, 125, 62.5 mg/mL using two fold broth dilution method. Cultures were then incubated at 37 °C for 24 h. MIC was determined as the lowest concentration of plant extract that completely suppressed the growth of microorganism (which is determined by the tube showing no turbidity), the tested bacteria were exposed to broth without the extracts as a control. Minimum bactericidal concentration (MBC) was determined by subculturing two loopfuls of the MIC tubes that showed no turbidity to nutrient agar plates. The MBC is identified by determining the lowest concentration of antibacterial agent that reduces the viability of the initial bacterial inoculum by ≥99.9%. Antibacterial agents are usually regarded as bactericidal if the MBC is no more than four times the MIC. Because the MBC test uses colony-forming units as a proxy measure of bacterial viability.

**Figure:**
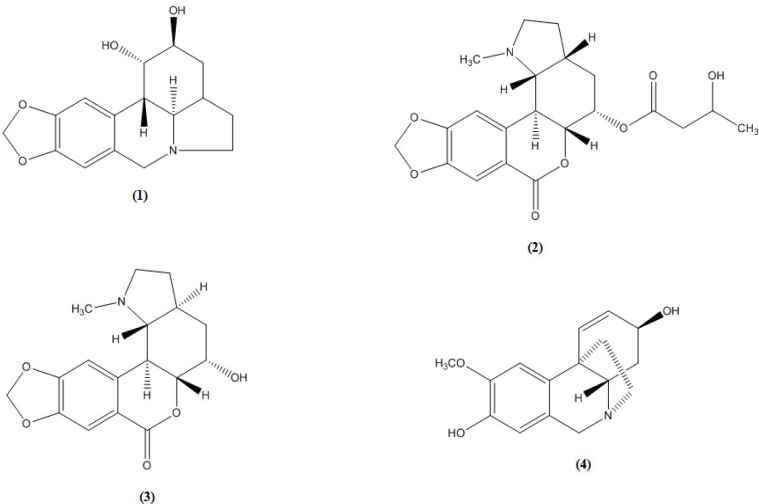


*Clivatine (2):*

 colourless crystals, mp: 175-177 °C,; IR υ_max_ cm^-1^: 3540, 1786, 1688; UV λ_max_, nm (abs.) MeOH: 308 (2.57), 268 (2.73), 236 (3.4); ^1^H NMR δ: 7.56 (1H, s, H-8), 7.02 (1H, s, H-11), 6.05 (2H, br-s, -OCH_2_O-), 5.04 (1H, m, H-5), 4.59 (1H, dd, H-5a, *J*_5a,11b_=11, *J*_5a,11c_ =10.5), 4.3 (1H, m, C-3’), , 3.35 (1H, dd, H-11b, *J*_11b,11c_=9.4, *J*_11b,5a_=11 Hz), 3.2 (1H, m, H-2*α*), 2.83 (1H, d, H-11c, *J*_11c, 11b_=9.4 Hz), 2.54 (1H, m, H-2*β*), 2.48 (H, m, H-3a), 2.45 (3H, s, Me-N), 2.35 (1H, d, C-2’, *J*=6), 2.2 (1H, m, H-4*α*), 2.1 (1H, m, H-3* α*), 2.04 (1H, m, H-4*β*), 1.93 (1H, m, H-3*β*), 1.3 (1H, d, C-4’, *J*=6).

*Nobilisine (3):*

colourless crystals, mp: 275-277 °C, IR υ_max_ cm^-1^: 3440, 1786, 1644; UV λ_max_, nm (abs.) MeOH: 295 (2.57), 260 (2.73), 226 (3.4); ); ^1^H NMR δ: 7.32 (1H, s, H-8), 7.1 (1H, s, H-11), 5.98 (2H, br-s, -OCH_2_O-), 4.33 (1H, dd, H-11b, *J*_11b,11c_=10 Hz), 4.1 (1H, d, H-5, *J*_5, 5a_=5.2 Hz), 3.94 (1H, dd, H-5a, *J*_5a,11b_ =11 Hz), 3.52 (1H, m, H-2*α*), 3.22 (1H, dd, H-11c,* J*_11c,3a_ =11.8 Hz, *J*_11c,11b_=10 Hz), 2.84 (1H, m, H-2*β*), 2.48 (H, m, H-3a), 2.35 (3H, s, Me-N), 2.19 (1H, m, H-4*α*), 2.13 (1H, m, H-3* α*), 1.98 (1H, m, H-4*β*), 1.95 (1H, m, H-3*β*).

*(+) 8-O-demethylmaritidine (4):*

colorless prisms*, *m.p. 160 °C; UV λ_max _nm (abs.) MeOH: 205.5 (1.3), 230 (0.26) 287(0.13); ^1^H-NMR δ: 6.77 (1H, s, H-10), 6.53 (1H, d, H-1, *J*_1,2_ = 9.8 Hz), 6.48 (1H, s, H-7), 5.86 (1H, dd, H-2, *J*_2__,1_ = 10.2, *J*_2,3 _= 5 Hz), 4.38 (1H, d, H-*6α, J*_6__α__,6__β _=16 Hz), 4.1 (1H, m, H-3), 3.8 (3H, s, -OCH_3_), 3.65 (1H, d, H-6*β, J*_6__β__,6__α_ =16 Hz), 3.38 (1H, dd, H-4a, *J*_4a,4__α_=12.7, *J*_4a,4__β_=4.6 Hz), 3.18 (1H, m, H-12*α*), 2.88 (1H, m, H-12*β*), 2.13 (1H, m, H-4*β*), 1.93 (1H, m, H-11*α*), 1.84 (1H, m, H-11*β*), 1.63 (1H, m, H-4*α*).

**Table 1:** Results of the antibacterial screening of the chloroform extract and some alkaloid isolates of *Clivia nobilis* flowers.

	**Inhibition zone (IZ) in mm** ^a^	
	**Gram-positive**	**Gram-negative**
	***Staphylococcus aureus***	***Pseudomonas aeroginosea***
Chloroform extract	17	20
Clivatine	18	25
Nobilisine	18	32
*8-O*-demethyl maritidine	23	28
Ciprofloxacin	0	30
Amoxicillin/ Clavulinic	30	0

a Values expressed are averages of three replicates.

**Table 2:** Minimum inhibitory concentration (MIC) and Minimum bactericidal concentration (MBC) of the chloroform extract and some alkaloid isolates of *Clivia nobilis* flowers

	***Staphylococcus aureus***		***Pseudomonas aeroginosea***	
	**MIC (μg/m** **l** **)**	**MBC (μg/m** **l** **)**	**MIC (μg/m** **l** **)**	**MBC (μg/m** **l** **)**
Chloroform extract	500	500	1000	1000
Clivatine	125	250	250	500
Nobilisine	31.25	62.5	62.5	125
*8-O*-demethyl maritidine	31.25	62.5	125	250

## Result and discussion

Four crystalline alkaloids were isolated from the flowers of *C. nobilis* cultivated in Egypt. They were identified as lycorine (1) with pyrrolo{de}phenanthridine nucleus (lycorine-type) which is the common alkaloid of the amaryllidaceae family, clivatine (2) nobilisine (3), both with [2] benzopyrano (3,4-g) indole nucleus (lycorenine-type) and (+) 8-*O*-demethylmaritidine (4) with 5,10b-ethanophenanthridine nucleus (crinine-type) using the available spectral data and by comparison with reference alkaloid samples (Co-chromatography and m.m.p.). 

The ^1^HNMR spectra of alkaloid (3) showed the presence of two singlets at 7.1 and 7.32 assigned respectively to H-11 and H-8 of the benzopyran moiety in agreement with the downfield effect induced on H-8 by the lactone group at C-7 (6, 9- 11).

It also showed two singlets typical of the methylenedioxy and the *N*-methyl groups resonating at 5.98 and 2.35 ppm respectively. The small coupling constants measured for H-5 with the two protons of C-4 and H-5a (*J*_5, 5a _= 5.2) allowed the assignment of an equatorial orientation for H-5 and axial orientation for the C-5 hydroxyl group and therefore the later assumes an *alpha *configuration.

Meanwhile, the large coupling constants between H-11b and both H-5a and H-11c indicate that the three protons assume an axial orientation and hence a *trans* B/C ring junction is indicated. Finally, the constant of 11.8 Hz measured for the coupling between H-11c and H-3a was consistent with an axial orientation of H-3a and therefore with a trans C/D ring fusion.

Alkaloid (3) was thus identified as nobilisine (6).

The ^1^H-NMR of alkaloid (2) differed from that of alkaloid (3) in the chemical shift value of H-5 which resonated at 5.04 ppm. This downfield shift suggests esterification of the C-5 hydroxyl group. This was confirmed from the appearance of a multiplet at 4.3 ppm which is coupled with the protons of a methylene (2.35, d, 2H) and a methyl (1.3, d, 3H) groups. In addition, some substantial differences were noted with respect to the stereochemistry of alkaloid (3), the C/D ring fusions should be different. The large typical values of the coupling constants between H-11b with both H-5a (J_5a,11b_=11 Hz) and H-11c (J_11b,11c_=9.4 Hz) together with the small values for the coupling of H-11c with H-3a determine an equatorial orientation for the latter proton indicating that the C/D ring fusion should be cis. Alkaloid (2) was thus identified as Clivatine (6, 11)

The presence of two singlets in the ^1^H-NMR spectra of alkaloid (4) at δ 6.77 and 6.48 assigned for the two aromatic protons H-10 and H-7, respectively, indicates disubstitution at C-8 and C-9 (12), while the appearance of signals for two olefinic protons, at δ 6.53 (d) assigned for H-1, and at δ 5.86 (dd) assigned for H-2, indicates the presence of double bond Δ^1,2 ^and a substituent at C-3. The presence of *α*-ethanobridge in addition to the quasi-axial *β*-oriented disposition of the C-3 hydroxyl were deduced from the coupling constants,* J*_2, 3_ = 5, *J*_3, 4__α_ =small undetetectable and the absence of allylic coupling between H-1 and H-3 (13). The large coupling between H-4a and H-4α (12.7 Hz) proves their trans/diaxial position characteristic for the haemanthamine series series (14-16). Each of the protons H-11*α*, H-11*β*, H-12*α* and H-12*β* is observed as ddd or m, which indicates a non-substituted ethanobridge (17). Material (4) is identified as (+) 8-*O*-demethylmaritidine (18, 19).

The results of antibacterial screening (Tables 1. and 2.) showed that the chloroform extract of the flowers of *C. nobilis* has antibacterial activity against the Gram-positive* Staphylococcus aureus* and the Gram-negative *Pseudomonas aeroginosea.* Meanwhile, the alkaloid nobilisine showed very good activity against the Gram-negative *P. aeroginosea *comparable to that of the standard used.
